# The diversity of isofrequency surface topologies in a hypercrystal composed of ferrite- and semiconductor-based metamaterials

**DOI:** 10.1038/s41598-023-43150-6

**Published:** 2023-09-26

**Authors:** Illia Fedorin

**Affiliations:** 1 Strategy and Innovations Department, Samsung R&D Institute Ukraine, Kyiv, Ukraine; 2 Biomedical Cybernetics Department, National Technical University of Ukraine “Igor Sikorsky Kyiv Polytechnic Institute”, Kyiv, Ukraine

**Keywords:** Metamaterials, Photonic crystals

## Abstract

Recent studies have centered on the potential for effectively controlling the topology state of iso-frequency surfaces in artificial photonic structures using external fields. This paper delves into the topological transitions and singularity states of the isofrequency surface of a highly anisotropic superlattice. This superlattice is composed of alternating layers of ferrite-dielectric and semiconductor-dielectric metamaterials. The superlattice is placed in an external magnetic field in the Voigt geometry that is parallel to the boundaries of the structure layers and perpendicular to the periodicity axis. Material properties of both constituent metamaterials are described in terms of effective components of permittivity and permeability in the long-wave approximation. An external magnetic field influences the properties of transverse electric (TE) waves in the ferrite-dielectric metamaterial, and the properties of transverse magnetic (TM) waves in the semiconductor-dielectric metamaterial. This results in the iso-frequency surface transition from a closed ellipsoid to an open hyperboloid for both TE and TM waves in various configurations. Furthermore, the superlattice can be identified as a hypercrystal under certain conditions, specifically when the constituent metamaterials possess a hyperbolic isofrequency surface state. This research demonstrates that the isofrequency surface properties of the studied hypercrystal can be effectively controlled by altering the external magnetic field, the fill factors of metamaterials, and frequency. Special attention is devoted to investigating the topological singularities that take place when iso-frequency surfaces of TE and TM polarized waves intersect. This intersection leads to the degeneracy of the hypercrystal’s isofrequency surface and the potential observation of unique phenomena such as conical refraction or the existence of surface states.

## Introduction

The study of the unique properties of isofrequency surfaces in various types of artificial systems, such as photonic crystals and metamaterials, is vital for comprehending the distinctive material properties of these systems, which are not immediately apparent from spectral properties and band structure analysis. Consequently, a deeper understanding of the physics behind various unique phenomena, such as negative refraction, superlens and superprism effects, and conical refraction can be achieved. A wealth of studies focused on examining the properties of photonic systems’ isofrequency surfaces has paved the way for the emergence of a new direction in solid-state physics, termed topological photonics^1–14^.

Depending on the sign and relative values of the permittivity and permeability tensor components in photonic structures, the topologies of isofrequency surfaces can manifest as closed ellipsoids or spheres, open hyperboloids, or various types of combinations of open and closed surfaces^5,7,9,13–16^. A key distinction among these various types of isofrequency surfaces lies in their ability to support the propagation of high-k electromagnetic modes common for hyperbolic materials with negative permittivity and/or permeability tensor components or the propagation of electromagnetic waves with limited wavevector values, typical for isotropic and anisotropic dielectrics with positive permittivity and permeability tensor components^1,12,14^. The ability to control both permittivity and permeability values, which are fundamental characteristics of photonic structures, presents new possibilities for the practical application of such structures or the discovery of fundamentally new states of light through achieving unique isofrequency surface states^3,8,17–25^.

Recent findings indicate that the tuning of constituent material parameters in artificial photonic structures may lead to bi-, tri- and tetrahyperbolic isofrequency surface topologies in bigyrotropic and bianisotropic materials^3–5,7–9,13,14,19^. Furthermore, the occurrence of Dirac-like singularities on the isofrequency surface of lossless epsilon-near-zero and/or mu-near-zero metamaterials, which is analogous to an effective zero optical mass, has generated significant interest. In such systems, phenomena such as giant optical non-locality, conical refraction, or unique surface states can be observed^26–31^.

In this paper, we investigate both theoretically and numerically the properties of the isofrequency surface of a superlattice, comprising alternating layers of ferrite-dielectric and semiconductor-dielectric metamaterials. Under certain material parameters, this superlattice can be considered as hypercrystal. Photonic hypercrystals are a novel class of artificial materials that synergize the most intriguing properties of photonic band gap crystals and hyperbolic metamaterials, facilitating the propagation of, theoretically, unlimited high-k waves^32–41^. The exceptional characteristics of these hypercrystals surpass the limited light emission capabilities of both photonic crystals and metamaterials and hold promising potential for novel applications in optoelectronics and radiophysics. By employing the effective medium theory, we derive the effective components of the permittivity and permeability tensors for both the hypercrystal and the constituent metamaterials. The entire system is subject to an external magnetic field applied parallel to the boundaries of the layers and perpendicular to the periodicity axis of the system. The resonant frequencies of the effective permittivity tensor for the semiconductor-dielectric metamaterial fall within the THz range, while those of the effective permeability tensor for the ferrite-dielectric metamaterial lie in the microwave range. The ranges of hyperbolicity and diabolic-like singularities of the isofrequency surface of the studied hypercrystal were revealed. The influence of the external magnetic field, frequency, and metamaterial filling factors as driving factors for topological transitions is discussed.

## Methodology

### Basic equations and system geometry

**Figure 1:**
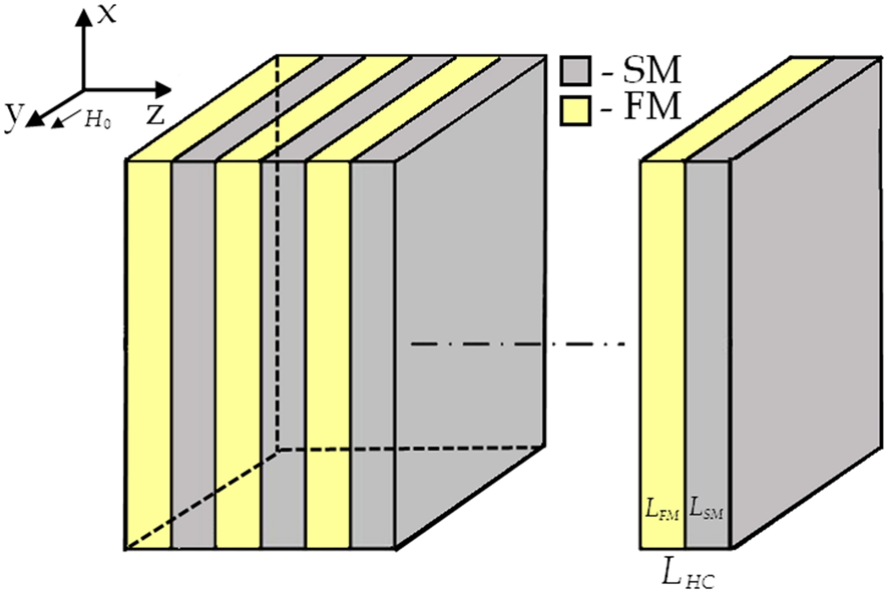
Geometry of the problem under study.

The hypercrystal (HC) under study is a periodic structure composed of alternating layers of two distinct types of metamaterials. The first layer of the HC unit cell comprises a ferrite-based metamaterial (FM), and the second layer is made up of a semiconductor-based metamaterial (SM). The system extends infinitely along the z-axis, which serves as the axis of periodicity, while the x-axis runs parallel to the layer boundaries (Fig. 1). The ferrite-based metamaterial is itself a periodic structure consisting of alternating layers of ferrite and dielectric materials. These layers have thicknesses of $d_f$ and $d_{d1}$, respectively, and the effective permittivity and permeability tensors of FM are represented as $\varepsilon ^{FM}$ and $\mu ^{FM}$. On the other hand, the semiconductor-based metamaterial consists of alternating layers of semiconductor and dielectric materials, with thicknesses of $d_s$ and $d_{d2}$, respectively. The tensors of effective permittivity and permeability of the SM are denoted as $\varepsilon ^{SM}$ and $\mu ^{SM}$. The total thickness of the HC unit cell is $L_{HC} = L_{FM} + L_{SM}$, which includes the FM thickness of $L_{FM} = N_{FM}d_{FM}$ ($d_{FM}$ is the period of FM, $N_{FM}$ is the number of FM periods), and the SM thickness of $L_{SM} = N_{SM}d_{SM}$ ($d_{SM}$ is the period of SM, $N_{SM}$ is the number of SM periods). The HC system is placed in an external magnetic field, which is applied parallel to the *y*-axis, running along the layers. The system is considered infinite along both the *x*- and *y*-axes. In the system under study, Maxwell’s equations split into independent sets for TM and TE polarizations, corresponding to $E_x$, $H_y$, $E_z$ and $H_x$, $E_y$, $H_z$ field components, respectively^32^.

Let us consider the system under a subwavelength approximation. This entails that all dimensions of the system, including the thicknesses of constituent layers in the metamaterials, the periods of the hypercrystal (HC), ferrite-based metamaterial (FM), and semiconductor-based metamaterial (SM), are significantly smaller than the wavelength of the propagating electromagnetic waves within the structure. Additionally, the thickness of the HC unit cell surpasses the periods of both the FM and SM structures. With this approximation, the material properties of the FM, SM, and HC can be captured in terms of effective medium parameters, allowing the application of a standard homogenization technique.

For the FM and SM metamaterials, the effective permittivity and permeability tensors can be presented as:

- for FM metamaterial
  1 $$\begin{aligned} \hat{\varepsilon }^{FM} = \begin{pmatrix} \varepsilon _{xx}^{FM} &{}\quad 0 &{}\quad 0 \\ 0 &{}\quad \varepsilon _{yy}^{FM} &{}\quad 0 \\ 0 &{} \quad 0 &{}\quad \varepsilon _{zz}^{FM} \\ \end{pmatrix}, \hat{\mu }^{FM} = \begin{pmatrix} \mu _{xx}^{FM} &{}\quad 0 &{}\quad 0 \\ 0 &{} \quad \mu _{yy}^{FM} &{} \quad 0 \\ 0 &{}\quad 0 &{}\quad \mu _{zz}^{FM} \\ \end{pmatrix}. \end{aligned}$$
- for SM metamaterial
  2 $$\begin{aligned} \hat{\varepsilon }^{SM} = \begin{pmatrix} \varepsilon _{xx}^{SM} &{}\quad 0 &{}\quad 0 \\ 0 &{} \quad \varepsilon _{yy}^{SM} &{}\quad 0 \\ 0 &{} \quad 0 &{}\quad \varepsilon _{zz}^{SM} \\ \end{pmatrix}, \hat{\mu }^{SM} = 1. \end{aligned}$$

The components of the corresponding tensors can be written as follows:

- for FM metamaterial
  3 $$\begin{aligned} \begin{aligned} \varepsilon _{xx}^{FM}&= \varepsilon _{yy}^{FM} = \frac{\varepsilon _{F}d_{f}+\varepsilon _{d1}d_{d1}}{d_{FM}}, \varepsilon _{zz}^{FM} = d_{FM}\left[ \frac{d_{d1}}{\varepsilon _{d1}}+\frac{d_{f}}{\varepsilon _{F}}\right] ^{-1};\\ \mu _{xx}^{FM}&= \frac{\mu _{F}d_{f}+d_{d1}}{d_{FM}}, \mu _{yy}^{FM} = 1, \mu _{zz}^{FM} = \frac{\mu _{xx}^{FM}\mu _{zz}^{*}}{\mu _{xx}^{FM}+\alpha \mu _{zz}^{*}},\\ \mu _{zz}^{*}&= \frac{d_{FM}\mu _{F}}{\mu _{F}d_{FM} + d_f}, \alpha = \frac{d_{f}d_{FM}}{d_{FM}^2\mu _{F}}\frac{\mu _{\bot }^2}{\mu _{\parallel }^2}. \end{aligned} \end{aligned}$$
- for SM metamaterial
  4 $$\begin{aligned} \begin{aligned} \varepsilon _{xx}^{SM}&= \frac{\varepsilon _{d2}d_{s} + \varepsilon _{f}d_{d2}}{d_{SM}}, \varepsilon _{yy}^{SM} = \frac{\varepsilon _{d2}d_{s} + \varepsilon _{yy}^sd_{d2}}{d_{SM}}, \\ \varepsilon _{zz}^{SM}&= \frac{\varepsilon _{xx}^{SM}d_{SM}^2}{d_{s}d_{d2}\left( \frac{\varepsilon _f}{\varepsilon _{d2}} + \frac{\varepsilon _{d2}}{\varepsilon _{\parallel }}\right) +d_{s}^2+d_{d2}^2}. \end{aligned} \end{aligned}$$

In the equations above $\varepsilon _{d1}$ and $\varepsilon _{d2}$ are the permittivities of the dielectric layers of FM and SM, respectively; $\varepsilon _F$ is the permittivity of the ferrite layer; $\mu _f$ is the ferrite layers effective magnetic permeability, $\mu _{\bot }$ and $\mu _{\parallel }$ are the components of the ferrite permeability tensor; $\varepsilon _f$ is the Voight effective permittivity of semiconductor layers, $\varepsilon _{\bot }$, $\varepsilon _{\parallel }$ and $\varepsilon _{yy}^s$ are the components of the semiconductor permittivity tensor. Detailed expressions for the corresponding components can be found in our previous publications^32,42,43^.

The FM metamaterial acts as a biaxial crystal with two distinct components of effective permittivity and three different components of effective permeability (considering that the *y*-axis component is frequency-independent and equal to 1). The anisotropy of the FM structure is primarily due to the anisotropy of the ferrite layer, which is reflected in the resulting anisotropy of effective permeability. The components of the effective permeability are highly dependent on the frequency and external magnetic field. Conversely, the effective permittivity is frequency and external magnetic field independent, and can only be adjusted by changing geometric factors ($\delta _{FM}^{f} = d_{f}/d_{FM}$ and $\delta _{FM}^{d1} = d_{d1}/d_{FM}$ are the ferrite and dielectric filling factors in the FM structure).

In contrast, the SM metamaterial is a biaxial crystal with three different principal components of effective permittivity tensor. The structure is non-magnetic in the sense that its permeability is a constant value, independent of frequency and magnetic field, and equal to 1. The anisotropy of the system in this case is entirely due to the anisotropy of the semiconductor material. Its permittivity becomes highly anisotropic in an external magnetic field and, thus, strongly dependent on frequency and external magnetic field ($\delta _{SM}^{s} = d_{s}/d_{SM}$ and $\delta _{SM}^{d2} = d_{d2}/d_{SM}$ are the semiconductor and dielectric filling factors in the SM structure).

In the subwavelength approximation, when considering the entire periodic structure, specifically, when the sum of the FM and SM thicknesses (representing the period of the system) is much smaller than the wavelength, it is likewise possible to introduce the following components of the effective permittivity and permeability of the HC:

5 $$\begin{aligned} \begin{aligned} \hat{\varepsilon }^{HC}&=\left( {\begin{matrix} {\varepsilon _{xx}^{HC}} &{} 0 &{} 0 \\ 0 &{} {\varepsilon _{yy}^{HC}} &{} 0 \\ 0 &{} 0 &{} {\varepsilon _{zz}^{HC}} \\ \end{matrix} } \right) ,~ \hat{\mu }^{HC}=\left( {\begin{matrix} {\mu _{xx}^{HC}} &{} 0 &{} 0 \\ 0 &{} 1 &{} 0 \\ 0 &{} 0 &{} {\mu _{zz}^{HC}} \\ \end{matrix} } \right) , \\ \varepsilon _{xx}^{HC}&= \varepsilon _{xx}^{FM}\delta _{FM} + \varepsilon _{xx}^{SM}\delta _{SM}, \varepsilon _{yy}^{HC} = \varepsilon _{yy}^{FM}\delta _{FM} + \varepsilon _{yy}^{SM}\delta _{SM},\\ \varepsilon _{zz}^{HC}&= \frac{\varepsilon _{zz}^{FM} \varepsilon _{zz}^{SM}}{\varepsilon _{zz}^{FM}\delta _{SM} + \varepsilon _{zz}^{SM}\delta _{FM}}, \mu _{xx}^{HC} = \mu _{xx}^{FM}\delta _{FM} + \mu _{xx}^{SM}\delta _{SM}, \\ \mu _{yy}&= 1, \mu _{zz}^{HC} = \frac{\mu _{zz}^{FM} \mu _{zz}^{SM}}{\mu _{zz}^{FM}\delta _{SM} + \mu _{zz}^{SM}\delta _{FM}}. \end{aligned} \end{aligned}$$

In (5) $\delta ^{FM} = L_{FM}/L_{HC}$ and $\delta ^{SM} = 1-\delta ^{FM}=L_{SM}/L_{HC}$ are the FM and SM filling factors, respectively.

Remarkably, since the permeability of the SM metamaterial is constant and equals 1, the permeability of the HC is mainly determined by the FM permeability tensor and only partially depends on SM parameters via the geometric factor. On the other hand, the permittivity of the HC is determined by the permittivity of both the SM and FM metamaterials. However, since the FM permittivity is frequency and external magnetic field independent, the properties of the resulting HC permittivity tensor are mainly determined by the SM permittivity and partially by the FM permittivity via the geometric factor.

The wavenumbers for TE and TM waves in the hypercrystal (HC) under study, following the homogenization procedure in the *xz* plane, can be expressed as follows:

6 $$\begin{aligned} \left( k_{z}^{TE} \right) ^{HC} = \sqrt{k_0^2\varepsilon _{yy}^{HC}\mu _{xx}^{HC} - \frac{\mu _{xx}^{HC}}{\mu _{zz}^{HC}}k_x^2}, \left( k_{z}^{TM} \right) ^{HC} = \sqrt{k_0^2\mu _{yy}^{HC}\varepsilon _{xx}^{HC} - \frac{\varepsilon _{xx}^{HC}}{\varepsilon _{zz}^{HC}}k_x^2}, \end{aligned}$$

In (6) $k_0 = \omega / c$. Thus, we can observe that the external magnetic field influences the properties of TE-waves in a ferrite layer solely through the *x* and *z* components of the effective FM permeability. Similarly, the properties of TM-waves in a semiconductor layer are affected only by the *x* and *z* components of the effective SM permittivity.

Detailed analysis of the FM, SM and HC permittivity and permeability tensors versus frequency, external magnetic field and geometric factor gives detailed insight into the dispersion properties of the system under study defining the topological transition points and corresponding ranges of hyperbolic and spherical isofrequency surface behavior along with new types of topological states and their combinations, which may give rise to topological singularity points.

### Frequency and external magnetic field dependence of effective permittivity and permeability tensor components

An in-depth study of the dielectric permittivity and magnetic permeability tensors of the FM, SM, and HC structures, with respect to frequency, external magnetic field, and constituent layer filling factors, can offer comprehensive insights into the dispersion properties of the system under study. By identifying points of topological transition, as well as regions exhibiting hyperbolic and ellipsoidal isofrequency surface behavior, it is possible to elucidate new types of topological states and their combinations. These insights may potentially lead to the discovery of topological singularity points.

Hereafter, all numerical calculations are made for the following system parameters (the same parameters as in our previous paper^32^):

1. FM metamaterial includes ferrite layers with relative dielectric permittivity $\varepsilon _{F} = 5.5$, saturation magnetization $M = 382G$, layer thickness $0.5\ \upmu$m (these parameters correspond to the brand 1SCH4, the polycrystalline nickel ferrite NiO $\text {Fe}_2\text {O}_3$); dielectric layers have relative dielectric constant $\varepsilon _{d1} = 4.0$ and layers thickness $0.5\ \upmu$m. The period of the FM unit cell is $1.0\ \upmu$m, total thickness of FM metamaterial is $10.0\ \upmu$m (number of periods is equal 10).
2. SM metamaterial represents semiconductor layers of n-type indium antimonide (n-InSb) with crystal lattice dielectric permittivity $\varepsilon _{0} = 17.8$, plasma frequency $\omega _{P} = 8 \times 10^{11}$ s$^{-1}$, layers thickness $0.5\ \upmu$m; dielectric layers have relative dielectric constant $\varepsilon _{d2} = 2.7$ and layers thickness $0.5\ \upmu$m. The period of the SM unit cell is $1.0 \mu$m, total thickness of SM metamaterial is $10.0\ \upmu$m (number of periods is equal 10).
3. Thickness of the HC unit cell is $20.0\ \upmu$m.

In further calculations we ignore losses in the semiconductor and ferrite layers (electron collision frequency and magnetic damping, correspondingly).

**Figure 2:**
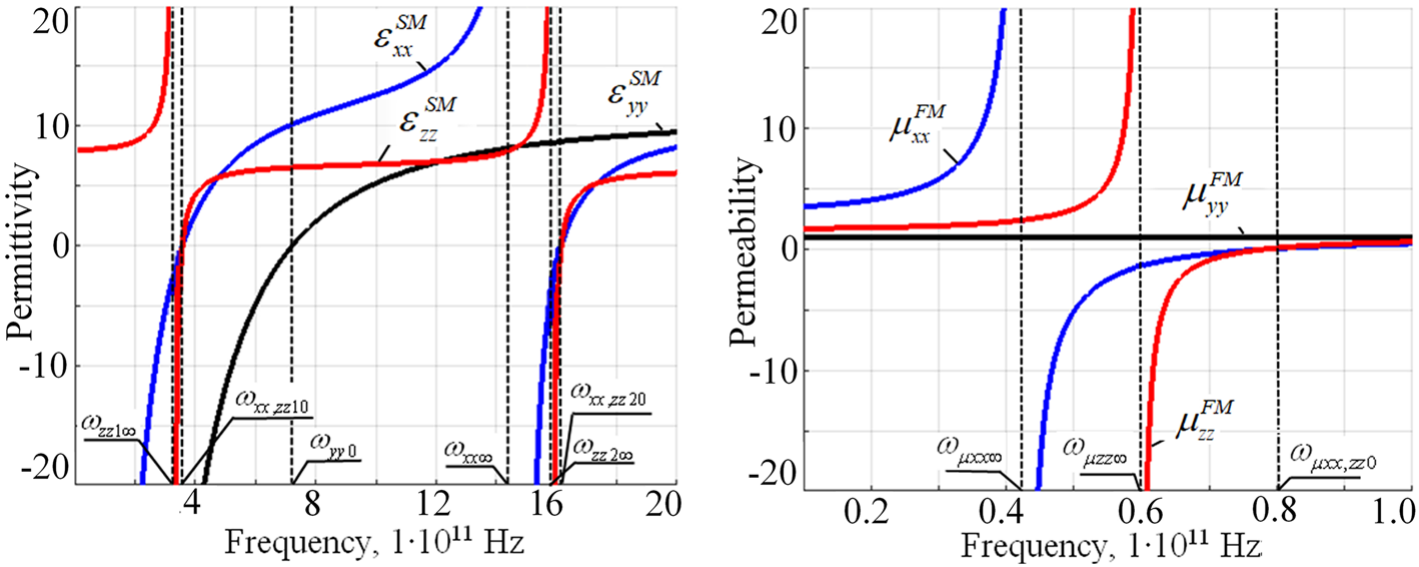
Frequency dependency of the FM permeability and SM permittivity tensor components ($H_0 = 1$ kOe).

The corresponding frequency dependency of FM permeability and SM permittivity is presented at the (Fig. 2). Several characteristic resonance frequencies and points can be defined at which metamaterials parameters become zero or tend to infinity in the idealistic lossless case. Hence, at these point FM permeability and SM permittivity tensor components change their sign and corresponding topological transition at the wave vector iso-frequency surface can occur.

Remarkably, characteristic frequencies of FM and SM metamaterials located far from each other at the frequency spectrum, namely in the microwave range for FM structure and in the THz range for SM structure. Thus, at the high frequency range above $\approx 2\times 10^{12}\ \text {s}^{-1}$ all components of SM permittivity and FM permeability are positive which should correspond to the closed spherical type of isofrequency surfaces. Below this frequency FM permeability and SM permittivity occur in different ratios in terms of their signs. At the frequency range below $\omega _{zz1\infty }$ (at which the SM permittivity component $\varepsilon _{zz}^{SM}$ tend to infinity) the SM permittivity tensor component $\varepsilon _{zz}^{SM}$ is positive, while $\varepsilon _{xx}^{SM}$ and $\varepsilon _{yy}^{SM}$ are strongly negative and all possible topological transitions may occur only in the FM metamaterial since its permeability experiencing a sign changes due to ferromagnetic resonance in the ferrite layers. Below the frequency $\omega _{\mu xx\infty }$ all components of the FM permeability are positive, while at the frequency range between $\omega _{\mu xx\infty }$ and $\omega _{\mu zz\infty }$: $\mu _{xx}^{FM}$ becomes negative; at frequency range between $\omega _{\mu zz\infty }$ and $\omega _{\mu xx,zz0}$: $\mu _{xx}^{FM}$ and $\mu _{zz}^{FM}$ are negative. In the frequency range between $\omega _{zz1\infty }$ and $\omega _{xx,zz20}$ all components of FM permeability are positive while in the SM permittivity tensor components are strongly influenced by hybrid and plasma resonances in the semiconductor layers which results in numerous ranges of permittivity sign changes. Thus, in the frequency range $\omega _{zz1\infty }$ and $\omega _{xx,zz10}$ all components of the SM permittivity are negative; in the frequency range between $\omega _{xx,zz10}$ and $\omega _{yy0}$ the SM permittivity components $\varepsilon _{xx}^{SM}$ and $\varepsilon _{zz}^{SM}$ are positive, while $\varepsilon _{yy}^{SM}$ is negative; in the frequency range $\omega _{yy0}$ and $\omega _{xx\infty }$ all components of the SM permittivity are positive; in the frequency range $\omega _{xx\infty }$ and $\omega _{zz2\infty }$ $\varepsilon _{xx}^{SM}$ is negative, while two other components are positive; in the frequency range $\omega _{zz2\infty }$ and $\omega _{xx,zz20}$ $\varepsilon _{xx}^{SM}$ and $\varepsilon _{zz}^{SM}$ are negative, while $\varepsilon _{yy}^{SM}$ is positive.

Such specific frequency dependence of permittivity and permeability tensor components in HC constituent materials gives rise to a unique topological states and a number of topological transitions in HC isofrequency surface. However, depending on geometrical factor (relative fraction of FM and SM metamaterial in the HC unit cell) specific features of isofrequency surfaces of FM and SM materials have different effects on HC properties.

**Figure 3:**
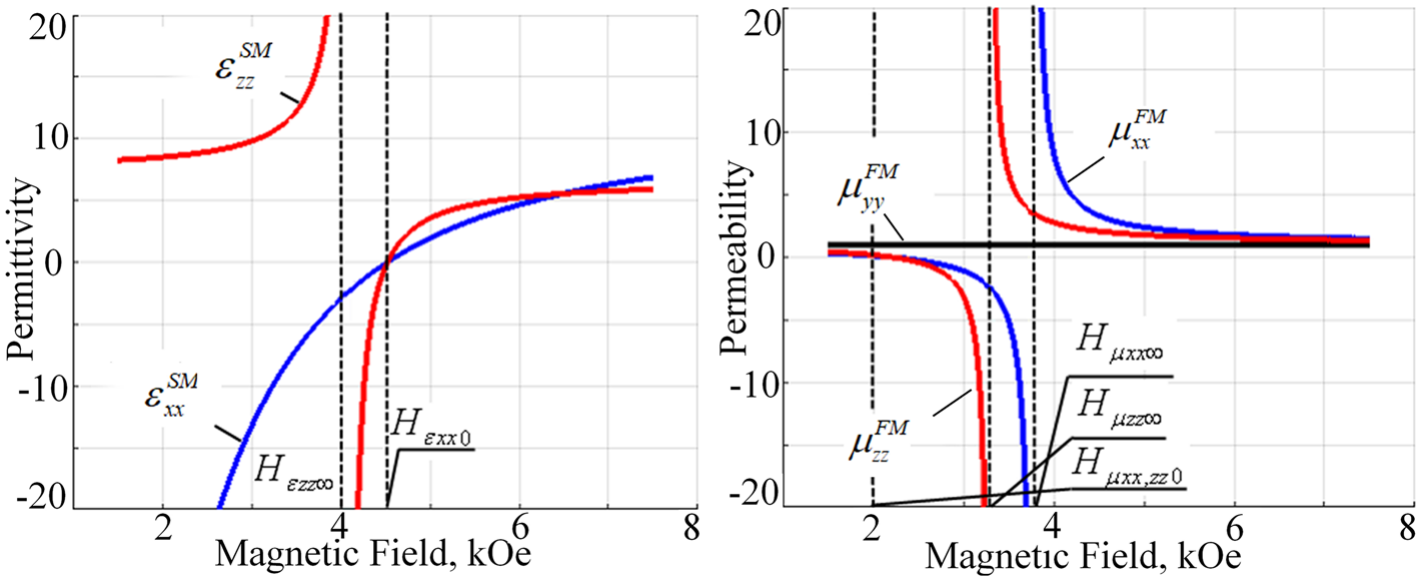
External magnetic field dependency of the FM permeability and SM permittivity tensor components ($\omega =1e11\ \text {s}^{-1}$).

Similar considerations hold for the SM and FM permittivity and permeability dependence’s versus external magnetic field (Fig. 3).

In this case the ferromagnetic resonance in the permeability tensor components of ferrite layers in the FM structure and hybrid resonance in permittivity tensor of semiconductor layers in the SM structure under the chosen materials parameters occur in the similar range of magnetic fields between 3 and 5 kOe, thus resulting in the corresponding numerous topological changes in this magnetic field range (Fig. 3). Thus, at low magnetic field values, lower approximately than 2 kOe (magnetic field $H_{\mu xx,zz0}$ at Fig. 3), all components of the FM permeability are positive; the SM permittivity component $\varepsilon _{xx}^{SM}$ is negative and $\varepsilon _{zz}^{SM}$ component is positive. At high magnetic fields, greater approximately 4.5 kOe (magnetic field $H_{\varepsilon xx0}$ at Fig. 3) all components of the SM permittivity and FM permeability are positive. Between those high and low magnetic field limiting values numerical changes in the signs of permittivity and permeability components takes place. Thus, between magnetic fields $H_{\mu xx,zz0}$ and $H_{\mu zz\infty }$ the FM permeability components $\mu _{zz}^{FM}$ and $\mu _{xx}^{FM}$ becomes negative; in the range between $H_{\mu zz\infty }$ and $H_{\mu xx\infty }$ the FM permeability component $\mu _{zz}^{FM}$ becomes positive again; after magnetic field $H_{\mu xx\infty }$ both $\mu _{zz}^{FM}$ and $\mu _{xx}^{FM}$ are positive. At little higher values of magnetic field, in the range between $H_{\varepsilon zz\infty }$ and $H_{\varepsilon xx0}$ both $\varepsilon _{zz}^{SM}$ and $\varepsilon _{xx}^{SM}$ components of SM permittivity are negative.

Detailed analytical expressions of the mentioned characteristic frequencies and magnetic fields can be found in our previous work^32^.

### Analysis of the HC effective permittivity and permeability tensor components

**Figure 4:**
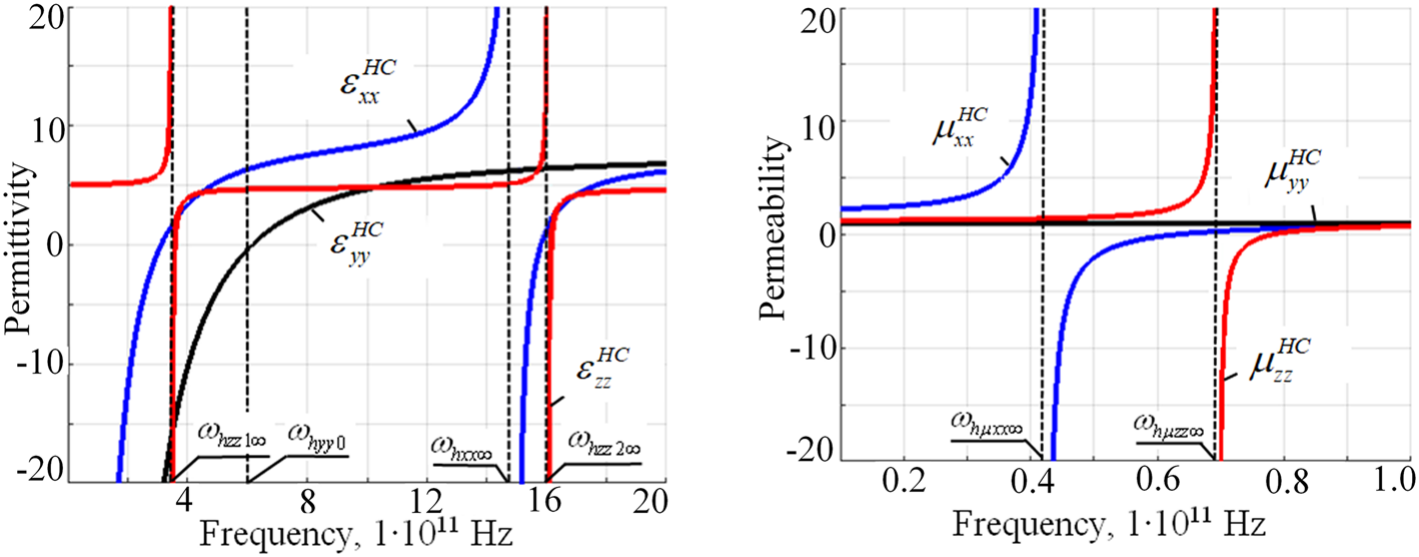
Frequency dependency of the HC effective permittivity and permeability tensor components ($H_0 = 1$ kOe, $\delta ^{FM} =\delta ^{SM}=0.5$).

**Figure 5:**
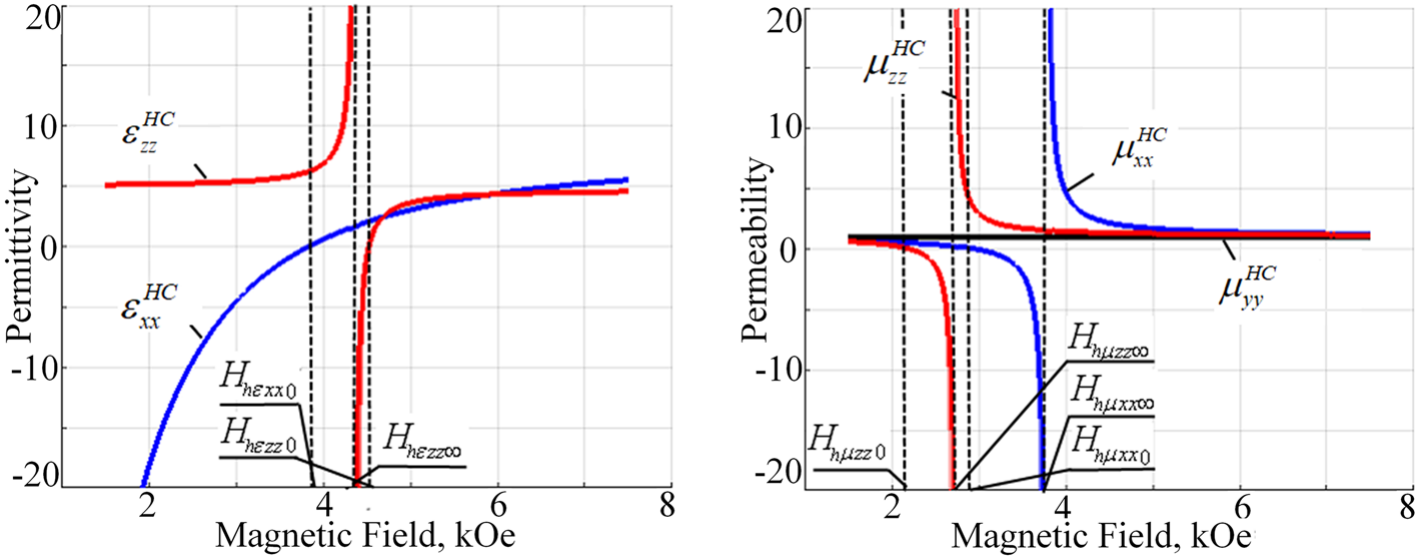
External magnetic field dependency of the HC permeability and permittivity tensor components ($\omega =1e11\ \text {s}^{-1}$, $\delta ^{FM} =\delta ^{SM}=0.5$).

**Figure 6:**
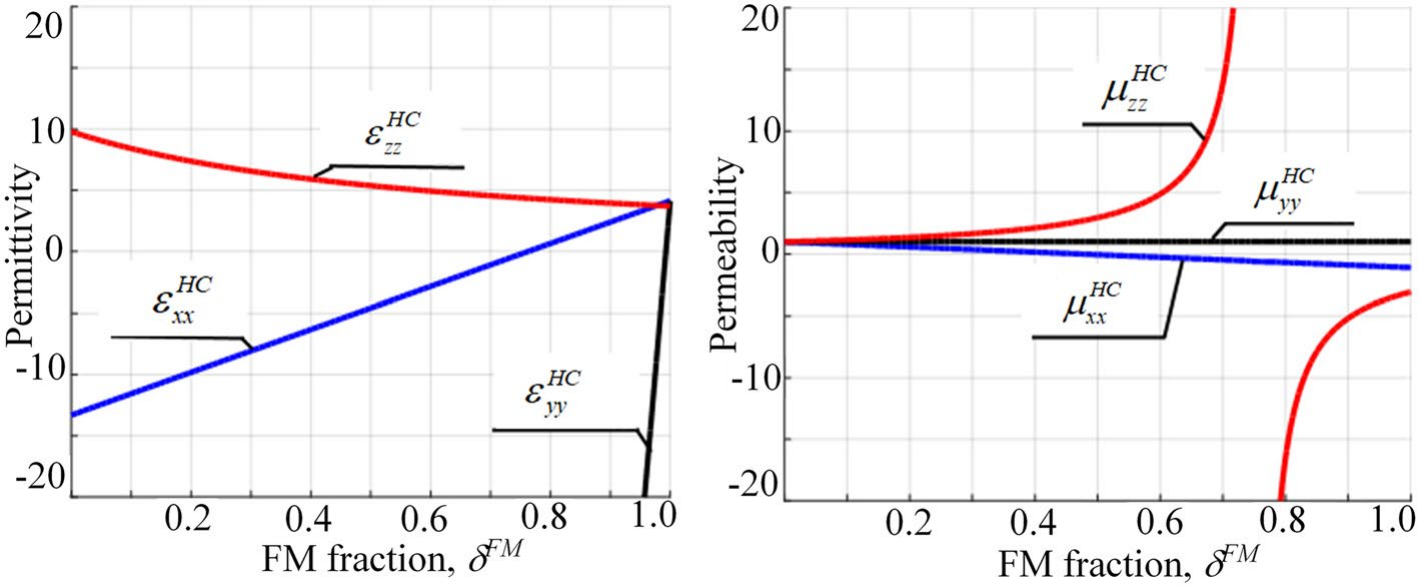
The HC effective permittivity and permeability versus FM and SM metamaterials thicknesses ($\omega =1e11\ \text {s}^{-1}$, $H_0=3$ kOe).

At Figs. 4, 5 and 6 the frequency, external magnetic field and filling factor dependencies of the HC under study are presented.

Considering the HC structure, it is likely that the effective properties of HC would be influenced by both frequency and magnetic field, similar to its constituent components, i.e., SM and FM metamaterials. The exact nature and extent of this influence would depend on the specific combination of the two constituent components in the HC, which is referred to as the “filling factor”.

The thicknesses of FM and SM layers within the HC unit cell significantly affect these dependencies. In particular, changing the FM and SM thicknesses modify the resonance frequencies of the materials, and therefore change the frequency and magnetic field ranges where specific phenomena, like sign changes of permittivity and permeability or topological transitions, occur. This alter the overall properties of the HC, as the effective permittivity and permeability of the HC are determined by the collective behavior of its components.

Frequency and external magnetic field dependencies of a HC effective permittivity and permeability components are presented at Figs. 4, 5. In a HC, both the FM and SM components contribute to the overall behavior, and the interplay between them can lead to complex effects that are not present in either of the individual components alone. This can include phenomena such as hybrid resonances, where the FM and SM layers interact to create resonances that are different from those of either component individually.

Increasing the thickness of the FM layers relative to the SM layers shift the effective properties of the HC towards those of FM, and vice versa. The influence of filling factor within HC is presented at the Fig. 6. The precise nature of this shift would likely be complex and would depend on a variety of factors, including the specific materials used, their geometrical arrangement, and the frequency and magnetic field conditions.

### HC isofrequency surface equation: equation of wavevector surface

Given that the HC under study effectively behaves as a biaxial crystal, its wave surface demonstrates a rather intricate geometry, comprised of two ellipsoidal surfaces interlaced with each other. When a section of this shape is made along the symmetry planes, the resulting figures are circles and ellipses. However, sections in other directions yield more complex shapes. The surface is shaped by two shells intersecting at four points and possesses a center of symmetry. Along two specific directions, known as optical axes or binormals, which pass through the origin and these intersecting points, the refractive indices ordinary and extraordinary waves align, leading to an absence of birefringence. For biaxial crystals, these optical axes stand perpendicular to the circular optical cross sections.

The principal values of the dielectric constant, which fluctuate depending on the frequency (wavelength), influence the orientations of the primary axes, and hence, the angle between them. This particular effect is termed as the dispersion of the optical axes, a characteristic primarily observed in monoclinic and triclinic crystals, particularly within the infrared spectrum.

Taking into account boundary conditions for tangential electromagnetic field components and using Maxwell equation, it is possible to derive the following wave normal surface equation for the structure under study (hereafter, for simplicity, the suffix HC is omitted):

7 $$\begin{aligned}&\left( \varepsilon _{xx}\mu _{xx}k_x^4+\varepsilon _{yy}\mu _{yy}k_y^4+\varepsilon _{zz}\mu _{zz}k_z^4 \right) + \left( \varepsilon _{xx}\mu _{yy}+\varepsilon _{yy}\mu _{xx}\right) \left( k_x^2k_y^2-\varepsilon _{zz}\mu _{zz}k_z^2k_0^2\right) +\\ &\quad \left( \varepsilon _{xx}\mu _{zz}+\varepsilon _{zz}\mu _{xx}\right) \left( k_x^2k_z^2-\varepsilon _{yy}\mu _{yy}k_y^2k_0^2\right) +\\&\quad \left( \varepsilon _{yy}\mu _{zz}+\varepsilon _{zz}\mu _{yy}\right) \left( k_y^2k_z^2-\varepsilon _{xx}\mu _{xx}k_x^2k_0^2\right) +k_0^4\varepsilon _{xx}\varepsilon _{yy}\varepsilon _{zz}\mu _{xx}\mu _{yy}\mu _{zz} = 0 \end{aligned}$$

For instance, when $k_y = 0$, the contour of the cross section of the given wave surfaces in the *zx* plane divides into two separate equations that are coincide with (6):

8 $$\begin{aligned} k_{z}^{TE} = \sqrt{k_0^2\varepsilon _{yy}\mu _{xx} - \frac{\mu _{xx}}{\mu _{zz}}k_x^2}, k_{z}^{TM} = \sqrt{k_0^2\mu _{yy}\varepsilon _{xx} - \frac{\varepsilon _{xx}}{\varepsilon _{zz}}k_x^2}, \end{aligned}$$

A similar situation arises in the cross sections of the two remaining coordinate planes. Under specific conditions, these two derived figures may intersect at four points. Lines drawn from the origin to each of these intersection points indicate the direction of the first kind of optical axes (also referred to as binormals or wave normal’s) of a biaxial crystal.

Therefore, by analyzing the wave surface equation and the iso-frequency surface of a HC, one can gain insights into the optical properties of the HC, as well as the conditions that lead to it exhibiting elliptic or hyperbolic behavior.

## Results and discussion

Hereafter, we are mainly focused on the topological states for different frequencies. In addition to the topological transitions illustrated across different frequencies, similar situation can also be induced by varying the external magnetic field or filling factor. The nature of these transitions lies in the modification of the relative values and signs of the effective permittivity and permeability tensor components, which can be influenced by frequency changes and magnetic field or filling factor adjustments. Unless otherwise specified, we will use the following parameters: $\omega =1e11\ \text {s}^{-1}$, $H_0 = 1$ kOe, $\delta ^{FM} =\delta ^{SM}=0.5$.

### Ranges of intersection of elliptic iso-frequency surfaces: conical refraction

Conical refraction is a unique optical phenomenon observed in certain anisotropic media, often associated with biaxial crystals, where an incident light wave splits into two concentric rings upon exiting the medium. This behavior can be understood in terms of the intersection of isofrequency surfaces of ordinary and extraordinary waves within the medium. The intersection forms a conical (ring-like) region in the momentum space, giving rise to the term conical refraction.

In the context of hyperbolic metamaterials such as HC under study, the phenomenon of conical refraction can potentially be tailored by manipulating various parameters of the system. These include the external magnetic field applied to the structure, the thicknesses of the FM and SM layers, etc. Adjustments in these parameters can lead to changes in the shape and orientation of the isofrequency surfaces, and hence, the properties and conditions under which conical refraction occurs.

**Figure 7:**
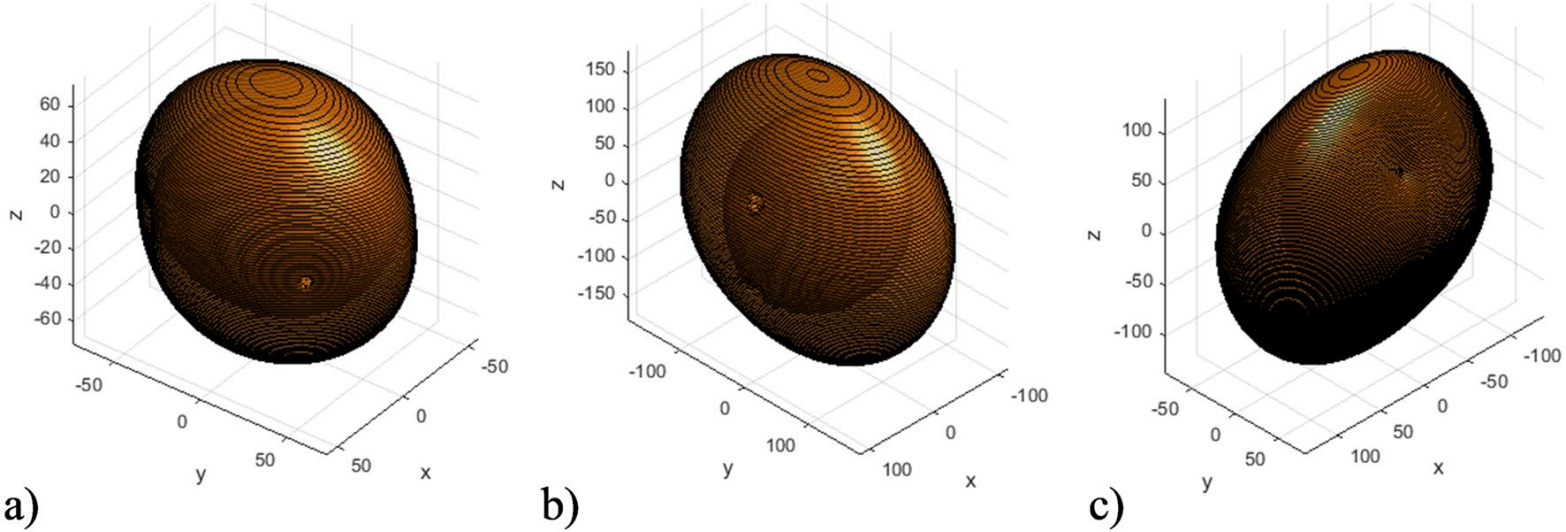
Ranges of elliptic isofrequency surface of the HC under different frequencies: (**a**) $\omega =8e11\ \text {s}^{-1} (\varepsilon _{xx}> \varepsilon _{zz} > \varepsilon _{yy})$; (**b**) $\omega =14e11\ \text {s}^{-1} (\varepsilon _{xx}> \varepsilon _{yy} > \varepsilon _{zz})$; (**c**) $\omega =16.2e11\ \text {s}^{-1} (\varepsilon _{yy}> \varepsilon _{zz} > \varepsilon _{xx})$.

Figure 7 presents the isofrequency surfaces of the considered HC at various frequencies with a fixed external magnetic field, resulting in different ratios between HC effective parameters. All components of effective permittivity and permeability at presented ranges are positive.

It can be observed that the relative values of the HC effective permittivity tensor components change for different frequencies. This results in changes to the HC isofrequency surface parameters, specifically the intersection planes of the ordinary and extraordinary waves. This ability to control conical refraction in such a system can open up new possibilities for manipulating light waves and could have potential applications in areas such as beam shaping, optical signal processing, and the development of novel optical devices.

### Ranges of hyperbolicity

Hyperbolic isofrequency surfaces are key characteristics of hyperbolic metamaterials and HC. They arise from the unusual behavior of these materials, which exhibit hyperbolic dispersion.

Hyperbolic dispersion means that the phase velocity of light (the speed at which the peaks and troughs of the wave propagate) can become infinite or even backwards, depending on the direction of propagation.

The isofrequency surfaces in these materials take the shape of hyperboloids. The exact nature of the isofrequency surfaces of such materials depends on the material properties and the frequency. By adjusting these factors, it is possible to manipulate the propagation of light for various applications.

#### Single hyperbolic surface

**Figure 8:**
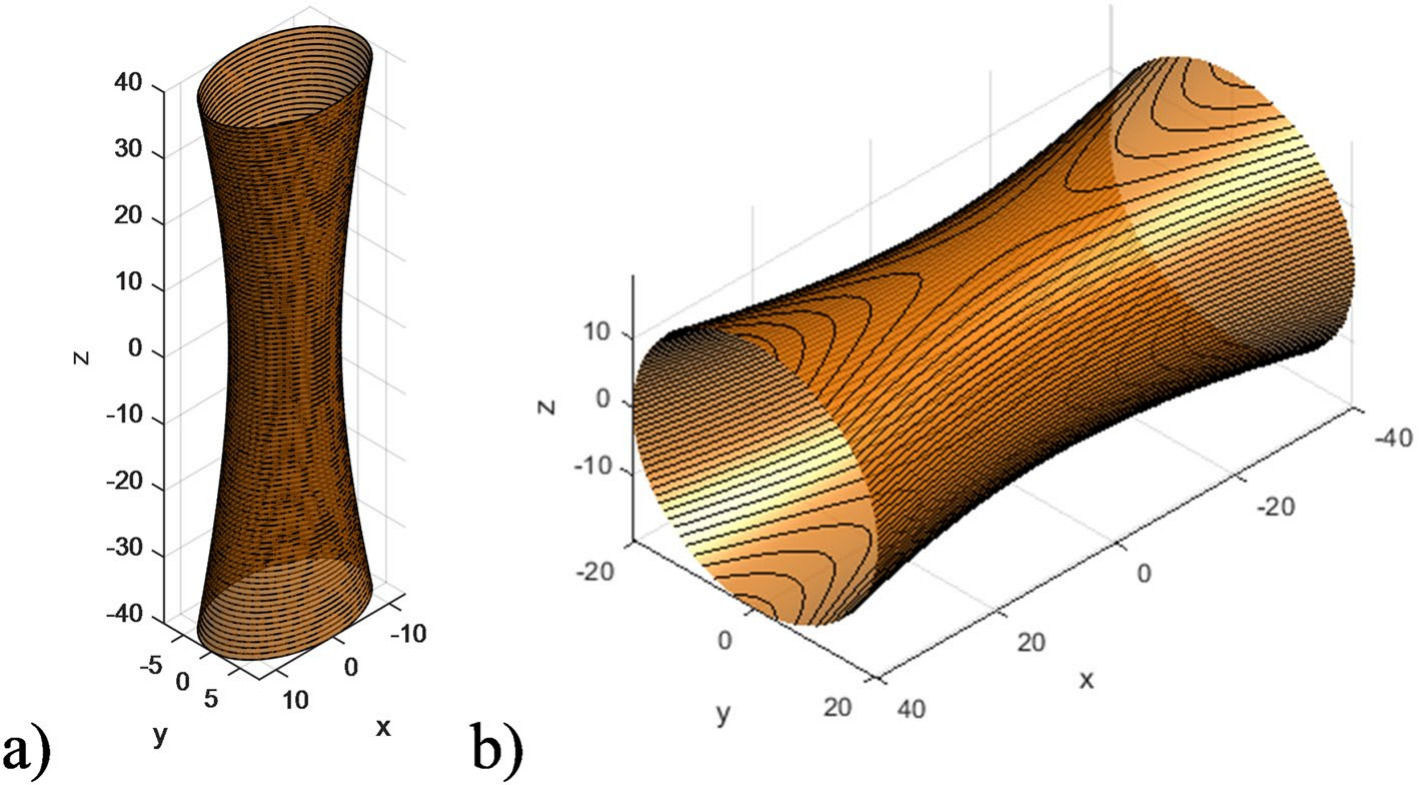
Ranges of singe hyperbolic isofrequency surface of the HC under different frequencies: (**a**) $\omega =1e11\ \text {s}^{-1} (\varepsilon _{zz} > 0; \varepsilon _{xx}, \varepsilon _{yy} < 0)$; (**b**) $\omega =3.5e11\ \text {s}^{-1} (\varepsilon _{xx} > 0; \varepsilon _{zz}, \varepsilon _{yy} < 0)$.

Figure 8 presents the isofrequency surfaces of the considered HC at various frequencies for the ranges where effective permeability tensor components are positive, whereas one component of effective permittivity ($\varepsilon _{xx}$ or $\varepsilon _{zz}$) is negative. This results in a single hyperbolic isofrequency surfaces directed along different axis.

It can be observed that, depending on the sign of the effective permittivity tensor components, the hyperbolic isofrequency surface represents a single hyperboloid. This hyperboloid extends infinitely along the *z*-axis when $\varepsilon _{zz} > 0$, and along the *x*-axis when $\varepsilon _{xx} > 0$.

#### Double hyperbolic surface

In cases when both the permittivity and permeability tensor components simultaneously become negative, the isofrequency surface of the HC can represent two hyperboloids, which extend infinitely along different axes depending on the relative values of the HC’s effective parameters.

**Figure 9:**
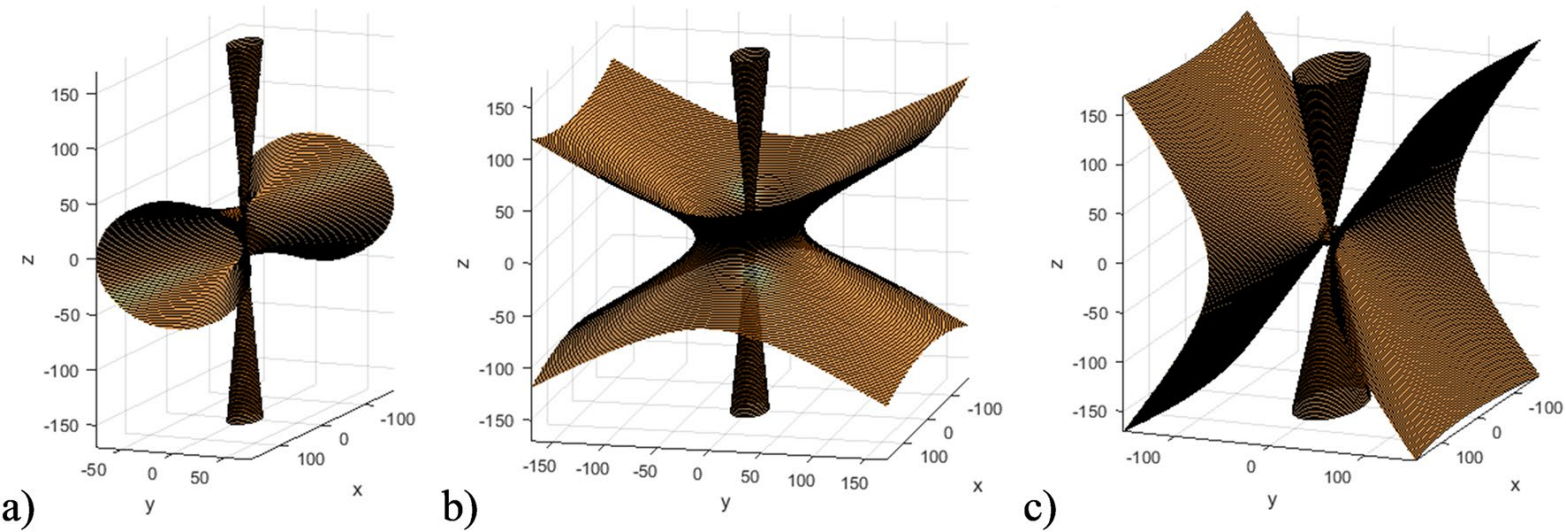
Ranges of double hyperbolic isofrequency surface of the HC under different frequencies (in all cases $\varepsilon _{zz} > 0$; $\varepsilon _{xx}$, $\varepsilon _{yy} < 0$): (**a**) $\omega =0.6e11\ \text {s}^{-1}$ ($\mu _{xx} < 0$); (**b**) $\omega =0.72e11\ \text {s}^{-1}$ ($\mu _{zz} < 0)$; (**c**) $\delta ^{FM} = 0.9$, $\delta ^{SM} = 0.1$ ($\mu _{xx} < 0$ and $\mu _{zz} < 0$).

Figure 9 presents the isofrequency surfaces of the considered HC at various frequencies for the ranges where both the effective permeability and permittivity tensor components may be negative. This results in double hyperbolic isofrequency surfaces directed along different axes.

It can be observed that, depending on the sign of the HC effective parameters, the hyperbolic isofrequency surface represents a double hyperboloid extending along different axes. Specifically, when $\mu _{xx}$ is negative, two hyperboloids are directed along the *x* and *z*-axes, while when $\mu _{zz}$ is negative, both hyperboloids extend infinitely along the *z*-axis. In the case when the fraction of FM material is predominant (as at Fig. 9c), both $\mu _{xx}$ and $\mu _{zz}$ are negative. This results in the isofrequency surface of the HC representing two hyperboloids, directed along the *y* and *z* axes.

The occurrence of two hyperboloids in the isofrequency surface can result in unique propagation characteristics. The two hyperboloids signify two different modes of wave propagation, each with their own unique properties. This can have implications for wave propagation, reflection, and transmission

### Ranges of combined elliptic and hyperbolic isofrequency surface

A combined elliptic and hyperbolic isofrequency surface typically occurs in complex materials, such as metamaterials and HC. In such materials, certain material parameters can lead to the occurrence of both elliptic and hyperbolic dispersion. The elliptic dispersion corresponds to regions where both the effective permittivity and permeability tensor components are positive, leading to a closed, elliptically-shaped isofrequency surface. On the other hand, hyperbolic dispersion occurs when one of the tensor components (either permittivity or permeability) is negative, resulting in an open, hyperbolically-shaped isofrequency surface.

These dual regions in the material’s frequency response can give rise to unique optical behaviors, including negative refraction and superlensing. The ability to switch between these states by adjusting parameters like the external magnetic field, thicknesses of layers, etc., offers exciting opportunities for tuning and manipulating the material’s electromagnetic response.

**Figure 10:**
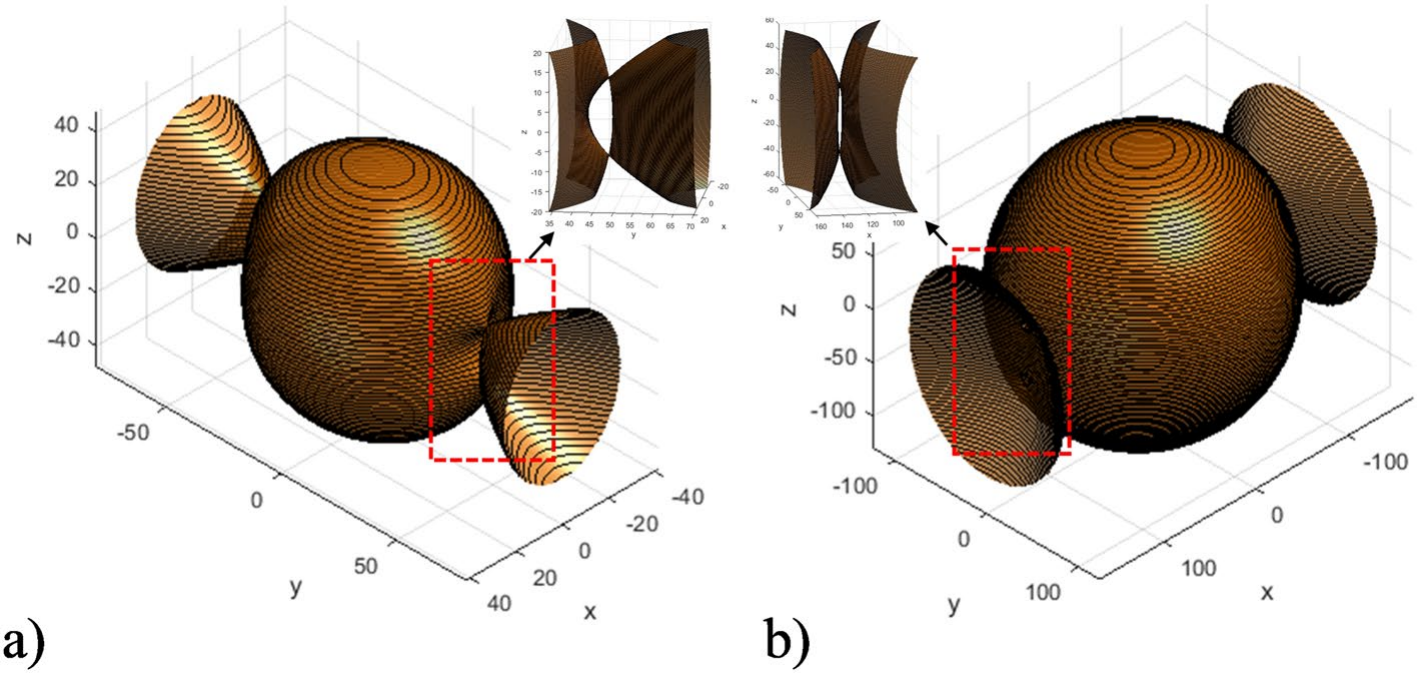
Ranges of dual elliptic and hyperbolic isofrequency surface of the HC under different frequencies: (**a**) $\omega =5.8e11\ \text {s}^{-1}$ ($\varepsilon _{zz}, \varepsilon _{xx} > 0; \varepsilon _{yy} < 0$); (**b**) $\omega =15.5e11\ \text {s}^{-1}$ ($\varepsilon _{zz}, \varepsilon _{yy} > 0; \varepsilon _{xx} < 0)$.

Figure 10 presents the isofrequency surfaces of the considered HC at various frequencies for the ranges where both elliptic and hyperbolic shapes coexist.

As observed in Fig. 10, for case *a*, where $\varepsilon _{zz}$ and $\varepsilon _{xx}$ are significantly positive and $\varepsilon _{yy}$ is negative, the HC isofrequency surface manifests as an intersection of an elliptically-shaped surface and a hyperbolic-shaped surface, with the latter extending along the *y*-axis. Similarly, for case *b*, where $\varepsilon _{zz}$ and $\varepsilon _{yy}$ are positive and $\varepsilon _{xx}$ is negative, the HC isofrequency surface also represents an intersection of elliptically-shaped and hyperbolically-shaped surfaces, this time with the hyperbolic surface extending along the *x*-axis.

In the specific case of the considered HC, the relative fractions of ferromagnetic (FM) and semiconductor (SM) materials in the HC unit cell, as well as their respective thicknesses, can also greatly influence the resultant isofrequency surface characteristics and the frequency ranges at which different dispersion regimes occur.

It is worth noting that the intersections between the ellipsoid and hyperboloid iso-frequency surfaces, as depicted in Fig. 10, serve as the geometric manifestations of the topological singularities discussed, fundamentally influencing the HC’s optical properties through localized field enhancements and novel wave-guiding capabilities.

### Ranges where HC isofrequency surface has different shape from constituent FM and/or SM materials

The ranges where the HC isofrequency surface takes on different shapes from its constituent ferromagnetic-based (FM) and/or semiconductor-based (SM) materials can occur under certain conditions, depending on the relative fractions and respective thicknesses of the FM and SM materials within the HC unit cell, frequency and applied external magnetic field values. This is due to the composite nature of the HC, which can exhibit unique collective behaviors not present in its individual components.

**Figure 11:**
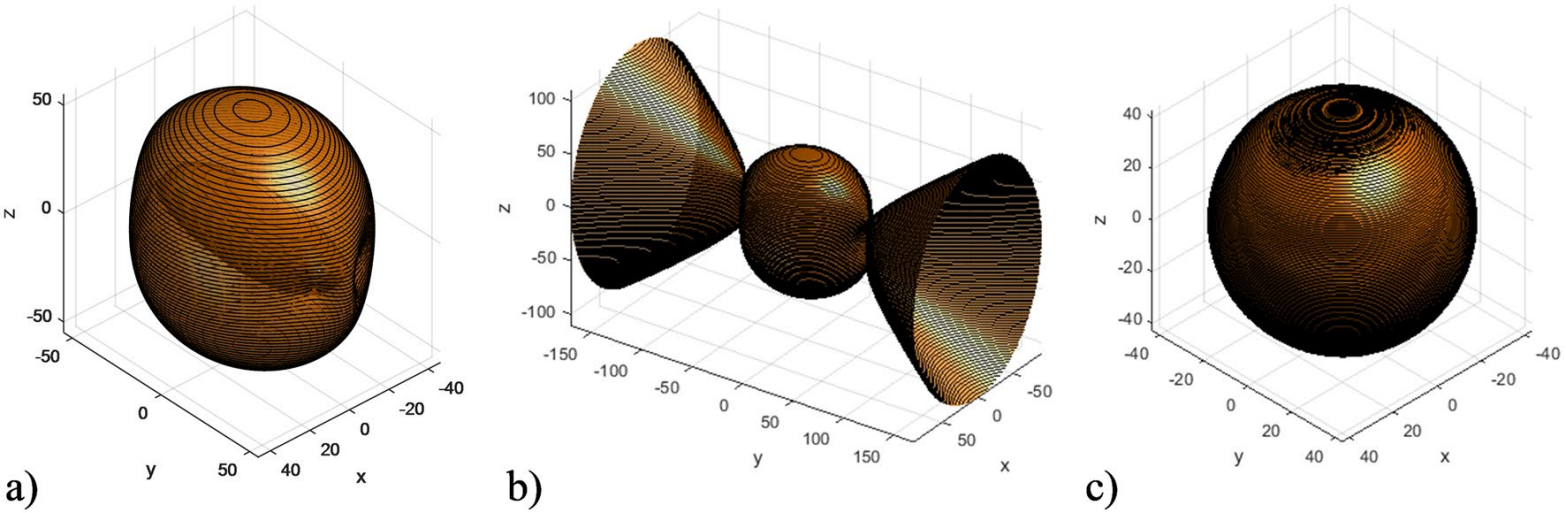
Example of the case, when the HC isofrequency surface diverges from those of the FM and / or SM materials ($\omega =6.4e11\ \text {s}^{-1}$): (**a**) HC isofrequency surface (all effective permittivity and permeability components are positive); (**b**) SM isofrequency surface ($\varepsilon _{yy} < 0$); (**c**) FM isofrequency surface (all permittivity and permeability components are positive).

As observed in Fig. 11, the isofrequency surface of the SM metamaterial represents combination of elliptical and hyperbolic shapes, due to the negativity of $\varepsilon _{yy}$ component of the SM effective permittivity tensor. At the same time, the resulting HC isofrequency surface is purely elliptical, since all components of the HC effective permittivity and permeability tensor components are positive.

These ranges, where the HC isofrequency surfaces diverge from those of the constituent materials, represent instances where the metamaterial displays properties that can be tailored for specific applications. They highlight the complex interactions of the HC’s effective permittivity and permeability tensor components, which can be influenced by changes in external factors such as frequency and magnetic field, as well as internal parameters like material composition and layer thicknesses. Understanding and leveraging these unique dispersion regimes could open up new possibilities in the design and application of metamaterials.

The case when all permittivity and permeability tensor components are simultaneously negative is not reachable under considered material parameters.

### Influence of losses

Above, all consideration were made for the lossless case. Losses in the semiconductor layer and magnetic damping in the ferrite layer are important factors to consider in a more comprehensive and real-world analysis. They introduce additional complexity to the behavior of the HC permittivity and permeability tensor components^44,45^.

Losses in the semiconductor layer. These losses are primarily due to absorption of electromagnetic waves, which converts the energy of the wave into heat. This can limit the effective transmission of the wave through the material. In the frequency domain, losses typically broaden the resonance and reduce the peak value, making the frequency response smoother.

Magnetic damping in the ferrite layer. This represents the loss of magnetic energy to the lattice of the material, which is then dissipated as heat. Similar to losses in the semiconductor layer, magnetic damping can smooth out resonances in the frequency response of the permeability.

Even with these loss mechanisms, however, it is still possible to observe sign changes in permittivity and permeability, leading to topological transitions in a real-word material. These changes might be less abrupt and occur at slightly different frequencies due to the broadening of the resonances. The general phenomena being studied, topological transitions due to changes in permittivity and permeability, should still occur, but the specifics would depend on the extent of the losses

## Conclusions

In conclusion, this paper presents a comprehensive study of a highly anisotropic superlattice, which is composed of alternating layers of ferrite-dielectric and semiconductor-dielectric metamaterials and is subjected to an external magnetic field. Under specific conditions related to the material parameters, this structure can be characterized as a hypercrystal. The ability to undergo topological transitions in hypercrystal isofrequency surface, transitioning from a closed ellipsoid to an open hyperboloid for both transverse electric (TE) and transverse magnetic (TM) waves, is studied.

The concept of the isofrequency surface of the composite structure is introduced, and studied how it changes with the material parameters. This led to the identification of distinct dispersion regimes, including elliptical, hyperbolic, and combined elliptical-hyperbolic states, each corresponding to different signs and combinations of effective material parameters. The study also identified instances where the composite’s isofrequency surface diverges from those of the constituent materials, thus highlighting the unique properties that arise from the hybrid structure.

In the synergy between ferrite and semiconductor-based metamaterials within a hypercrystal, distinct topological characteristics arise, setting it apart from standalone metamaterials. Specifically, the hypercrystal under study has the potential to manifest a bi-hyperbolic iso-frequency surface, where both the TE and TM wave surfaces concurrently adopt a hyperbolic shape. Such a phenomenon is unattainable in isolated FM or SM due to their differing magnetic field responses. A compelling consequence of this interplay is the ability to adjust the orientations of the hyperboloids by varying the frequency, magnetic field, and filling factor. This gives rise to diverse configurations, including their intersection, leading to unique singularity points unobserved in standalone composed metamaterials.

Thus, through this systematic study of the composite system, critical insights into the interplay between material composition, structural parameters, and external influences in shaping the effective electromagnetic properties is gained. The results may prove useful for designing and optimizing metamaterial-based devices across various frequency ranges, from microwaves to terahertz. Particularly, the findings underscore the potential for manipulative control over wave dispersion and propagation characteristics. This suggests potential applications in areas such as communications, sensing, and imaging, where metamaterials are commonly used

## Data Availability

The datasets used and/or analysed during the current study available from the corresponding author on reasonable request.
